# Clinical phenotype and outcome of persistent SARS-CoV-2 replication in immunocompromised hosts: a retrospective observational study in the Omicron era

**DOI:** 10.1007/s15010-023-02138-0

**Published:** 2023-12-14

**Authors:** Veronika Götz, Philipp Mathé, Prerana Agarwal, Daniel Hornuss, Stefanie Pfau, Marcus Panning, Eric Prager, Reinhard E. Voll, Monika Engelhardt, Björn C. Frye, Fabian Bamberg, Jonas Fuchs, Matthias Müller, Dirk Wagner, Siegbert Rieg

**Affiliations:** 1 Faculty of Medicine, Division of Infectious Diseases, Department of Medicine II, Medical Center, University of Freiburg, Hugstetter Str. 55, 79106 Freiburg, Germany; 2 Faculty of Medicine, Department of Radiology, Medical Center, University of Freiburg, 79106 Freiburg, Germany; 3 Faculty of Medicine, Institute of Virology, Medical Center, University of Freiburg, 79106 Freiburg, Germany; 4 Faculty of Medicine, Department of Nephrology, University Medical Center, University of Freiburg, 79106 Freiburg, Germany; 5 Faculty of Medicine, Department of Rheumatology and Clinical Immunology, Medical Center, University of Freiburg, 79106 Freiburg, Germany; 6 Faculty of Medicine, Department of Internal Medicine I, Hematology, Oncology and Stem Cell Transplantation, University Medical Center, University of Freiburg, 79106 Freiburg, Germany; 7 Faculty of Medicine, Department of Pneumology, Medical Center, University of Freiburg, 79106 Freiburg, Germany; 8 Department of Infection Medicine, Medical Service Centre Clotten, 79106 Freiburg, Germany

**Keywords:** SARS-CoV-2, Immunosuppression, Omicron, Lower respiratory tract, Antiviral therapy, Fibrotic-like lung changes

## Abstract

**Purpose:**

This study aims to describe clinical, virological and radiological characteristics as well as treatment strategies and outcomes of immunocompromised patients with persistent SARS-CoV-2 replication.

**Methods:**

We performed a retrospective cohort study of immunocompromised patients at the University Medical Center Freiburg between 01/2022 and 05/2023. Patients with substantial immunosuppression and persistent SARS-CoV-2 detection (Ct-value < 30 after 14 days) were included.

**Results:**

36 patients in our cohort reported mainly fever, dyspnoea or continuous cough. Viral load was significantly higher in concurrent samples taken from the lower respiratory tract (Ct-value = 26) than from the upper respiratory tract (Ct-value = 34). Time of detectable viral RNA after start of antiviral treatment was shorter in patients receiving two antivirals (median 15 days vs. 31 days with one antiviral agent). Short-course antiviral therapy (≤ 5 days) was less efficient in reduction of symptoms and viral load than prolonged therapy > 10 days. In 30% (8/27) of patients with repeated CT scans, we found the emergence of chronic pulmonary changes, which were more frequently in patients with B cell depletion (37%, 7/19) compared to patients with organ transplantation (12%, 2/17).

**Conclusion:**

Ongoing SARS-CoV-2 replication in the lower respiratory tract is a relevant differential diagnosis in patients with severe immunosuppression and continuous cough, fever or dyspnoea even if nasopharyngeal swabs test negative for SARS-CoV-2. Especially in B cell-depleted patients, this may lead to inflammatory or fibrotic-like pulmonary changes, which are partially reversible after inhibition of viral replication. Antiviral therapy seems to be most effective in combination and over a prolonged period of time of > 10 days.

**Trial registration number:**

DRKS 00027299.

**Supplementary Information:**

The online version contains supplementary material available at 10.1007/s15010-023-02138-0.

## Introduction

In the early phase of the SARS-CoV-2 pandemic with predominance of Alpha, Beta and Delta variants, immunocompromised patients were at risk to suffer from fulminant and often fatal courses of SARS-CoV-2 infection. With the global emergence of the Omicron variant and the broad availability of vaccinations, hospitalisation and mortality rates were declining [1]. Recent evidence mainly derived from case reports or case series points towards a change in the clinical manifestation of SARS-CoV-2 infection in immunocompromised patients from fulminant courses to long-term viral replication [2–11]. Yet, little is known about the clinical course of immunocompromised patients as well as long-term outcomes including chronic pulmonary changes. As most of these patients, especially after B cell depletion, are unable to mount a robust humoral immunity after vaccination or infection, effective antiviral treatment seems essential. However, efficacy of antiviral substances has not been studied in this patient subgroup and no general recommendations are available. Therefore, therapeutic management remains challenging [12].

In the Omicron era, a vast majority of patients treated for SARS-CoV-2 infection in our tertiary care centre suffer from underlying disease and most are immunocompromised. We present data from 36 patients with substantial immunosuppression and persisting SARS-CoV-2 detection. We aim to describe the clinical, virological and radiological characteristics as well as the management and outcome of immunocompromised patients with persistent SARS-CoV-2 replication in the Omicron era.

## Methods

### Patients and setting

We performed a retrospective cohort study of immunocompromised patients at the University Medical Center Freiburg (UMCF) between January 2022 and May 2023. This time period is characterised by the dominance of the Omicron variant and its sub-strains in our region [13]. The recruitment of patients was done by the Infectious Diseases (ID) consultation service (see Supplementary Fig. 1). Patients were included if a) substantial immunosuppression was present (see definitions) and b) SARS-CoV-2 with a Ct-value of < 30 (reflecting a concentration of around > 30.000 copies/ml in our setting) was detected in a respiratory sample > 14 days after the first positive test for SARS-CoV-2 [14–16].

### Definitions

Substantial immunosuppression was defined as patients receiving either immunosuppressive medication for solid organ transplants or having received B cell-depleting agents less than one year ago (like rituximab, obinutuzumab).

The start of the SARS-CoV-2 infection was defined as the first date with a positive SARS-CoV-2 PCR. In cases where only a time period for a positive SARS-CoV-2 PCR could be assumed based on clinical documentation, the date was chosen which led to the shortest possible time of persistent infection (3/36 cases).

Mode of acquisition was classified as hospital-acquired if the first detection of SARS-CoV-2 and its related symptoms occurred > 48 h after admission [17]. Recurrent fever was defined as body temperature > 38 °C more than 14 days after start of infection with an initial improvement of symptoms.

Treatment failure was defined by a persistent positive SARS-CoV-2 PCR (Ct-value < 40) > 14 days after end of performed therapy. Clinical treatment success was defined accordingly, namely as the absence of symptoms > 14 days after end of therapy.

### Data acquisition

Clinical data were retrieved from electronic health care records, including radiological and virological data. Due to official regulations, a random subgroup of patient samples were sequenced for variant detection as previously described [18]. SARS-CoV-2 PCR was performed according to standard protocols from naso-oropharyngeal swabs or broncho-alveolar lavage using CE in vitro-certified diagnostic assays.

### Radiological analysis

All of the acquired CT scans were retrospectively analysed in a dedicated reading session by an experienced thoracic radiologist (P.A.). The images were reviewed on a Picture Archiving and Communication System (PACS), using 1 mm slice thickness in standard lung window.

For the initial CT scan, the pattern of lung involvement was classified as per the RSNA Expert Consensus Statement and the CO-RADS classification systems, which suggest the level of suspicion of SARS-CoV-2 pneumonia [19]. Furthermore, the extent of overall lung involvement was scored semi-quantitatively with a score from 0 to 15 (volume of involvement of each lobe was scored as 1 when less than 1/3rd of parenchyma was involved, 2 for involvement of 1/3rd to 2/3rd of lobar volume and 3 when more than 2/3rd of the lobar volume was affected) [20]. A note was made of the axial and craniocaudal distribution of lung involvement. The predominant pneumonia pattern (ground-glass opacities, consolidation, or mixed) was analysed. In the follow-up CT scans, the extent of findings was compared to each previous CT for progression or regression and the residual findings were further characterised. Fibrotic-like changes referred to perilobular bands, bronchial dilatation and reticulations [21].

### Statistical analysis

For the comparison of viral load in broncho-alveolar lavage and naso-oropharyngeal swabs (the latter ± 2 days around the date of the lavage), a paired Wilcoxon rank-sum test was used. Fisher’s Exact test was used for group comparison of symptoms. Figures were produced using R Studio (R version 4.2.2).

## Results

We included 36 patients in our cohort (solid organ transplantation *n* = 16, B cell depletion *n* = 18, combined = 1, allogeneic stem cell transplantation *n* = 1). The main indication for B cell-depleting therapy was haematological malignancy (63%, 12/19). In 50% of patients (18/36), sequencing of virus was performed which revealed predominantly BA sub-variants (67%, 12/18), followed by BF sub-variants (17%, 3/18). Serological analysis of Anti-S1-antibody levels prior to infection showed a negative result for most of the patients (see Supplemental Fig. 5). Further cohort characteristics are displayed in Table 1.

**Table 1:** Cohort description

Variable	*n* (%)	Median (1.Q.–3.Q.)
*n* total	36	
**Patient characteristics**		
Age, years		63 (57–71)
Sex		
*Female*	13/36 (36%)	
*Male*	23/36 (64%)	
Vaccinated	31/34 (91%)	
Number of vaccination doses		4 (3–4)
Immunosuppression		
*Organ transplantation*^A,C^	17/36 (47%)	
*Kidney*	11/17 (65%)	
*Lung*	4/17 (24%)	
*Heart*	1/17 (6%)	
*Liver*	1/17 (6%)	
*B cell depletion*^B,C^	19/36 (49%)	
Reason for B cell depletion		
*Haemato-oncological malignancy*	12/19 (63%)	
*Rheumatological disease*	7/19 (37%)	
*Allogeneic stem cell transplantation*	1/36 (3%)	
Comorbidities		
*COPD*	1/36 (3%)	
*Other lung disease*	10/36 (28%)	
*Coronary heart disease/myocardial infarction*	9/36 (25%)	
*Heart failure*	7/36 (19%)	
*Active solid tumour*	1/36 (3%)	
*Active haemato-oncological malignancy*	16/36 (44%)	
*Diabetes*	7/36 (19%)	
*Chronic kidney disease*	25/36 (69%)	
*Chronic liver disease*	3/36 (8%)	
*Neurological disease*	10/36 (28%)	
*Number of comorbidities, n*		2 (2–3)
*Prophylaxis with Tixagevimab/Cilgavimab*	3/30 (10%)	
**Infection characteristics**		
Mode of acquisition		
*Community-acquired*	31/36 (86%)	
*Hospital-acquired*	5/36 (14%)	
Admission due to Covid-19	28/36 (78%)	
**Diagnostics**		
CT scan performed	27/36 (75%)	
Multiple CT scans performed	20/36 (56%)	
Bronchoscopy + BAL performed	15/36 (42%)	
**Clinical characteristics**		
Admitted to ICU	7/32 (19%)	
Low-flow oxygen	13/25 (52%)	
High-flow oxygen	7/31 (23%)	
Mechanical ventilation	2/32 (6%)	
In-hospital mortality	1/36 (3%)	
**Therapy**		
Additional Steroids	9/36 (25%)	
Antiviral agent used	29/36 (81%)	
*Nirmatrelvir/Ritonavir*	13/36 (36%)	
*Molnupiravir*	11/36 (31%)	
*Remdesivir*	18/36 (50%)	
Monoclonal antibody therapy	21/36 (58%)	
*Tixagevimab/Cilgavimab*	7/36 (19%)	
*Sotrovimab*	16/36 (44%)	
Combined antiviral therapy	11/36 (31%)	
Combined antiviral + antibody therapy	14/36 (39%)	
Antiviral therapy duration, days		5 (5–11)
Antiviral therapy duration > 5 days	9/36 (25%)	
Episodes of antiviral therapy		1 (1–2)

A: used agents included tacrolimus, corticosteroids, mycophenolate mofetil, ciclosporin, everolimus, azathioprine, siltuximab

B: beside rituximab and obinutuzumab additional used agents included corticosteroids, cyclophosphamide, anakinra, hydroxychloroquine, methotrexate, ibrutinib, ruxolitinib

C: One patient had treatment with B cell-depleting agent and solid organ transplantation and is therefore included in both groups

Abbreviations: *BAL*  Broncho-alveolar lavage, COPD  chronic obstructive pulmonary disease,* CT*  Computer tomography,* ICU* Intensive Care Unit,* STEMI*  ST elevation myocardial infarction

### Symptoms of patients

Cough (24/36 patients), fatigue (20/36), fever (18/36) and dyspnoea (14/36) were the predominant symptoms in the initial phase, with around 28% of patients (10/36) reporting all four symptoms. Prolonged or recurrent fever was found in 50% (18/36, of whom 6 were without fever at onset), 39% with dyspnoea (14/36, of whom 5 were without dyspnoea at onset), 44% with cough (16/36, of whom 3 were without cough at onset) and 47% with fatigue (17/36). Patients with B cell depletion more frequently reported symptoms than patients after solid organ transplantation (recurrent fever [79% vs. 24%, p < 0.001], dyspnoea [47% vs. 29%, ns], cough [42% vs. 41%, ns] or fatigue [53% vs. 47%, ns], respectively). Antibody levels after infection or vaccination were in median below the detection limit, with no significant differences between patients with B cell depletion or organ transplantation.

Nearly half of the patients needed low level oxygen via nasal cannula at initial presentation, yet, oxygen supplementation could be stopped before hospital discharge in all patients (excluding one patient with previous long-term oxygen therapy).

### Length and compartmentalisation of viral detection

**Fig. 1:**
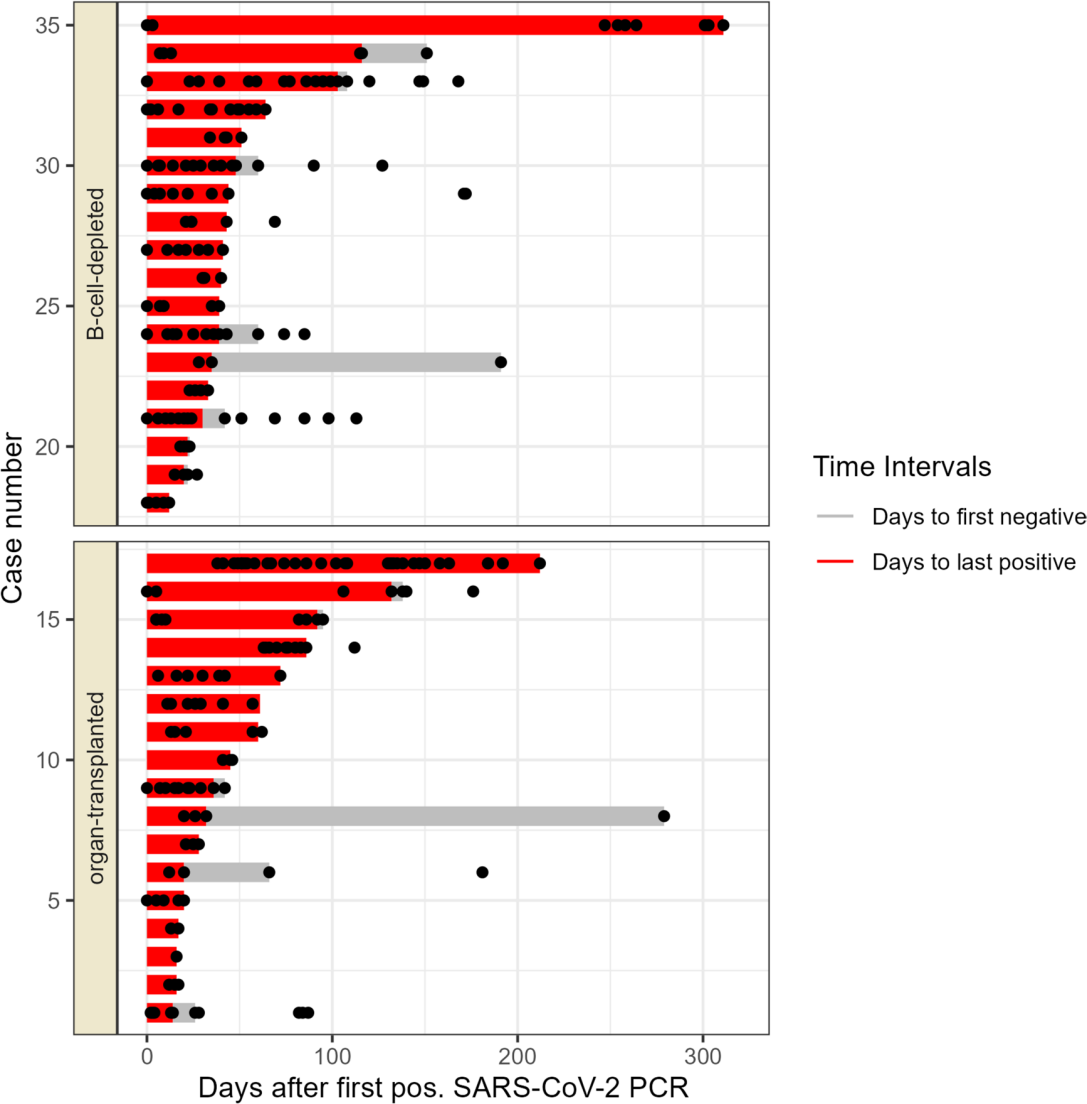
Length of SARS-CoV-2 positivity in patients. Black dots represent the time points when SARS-CoV-2 PCRs were performed. Median number of days between first positive and last positive sample according to group: B cell-depleted: 41 days, organ-transplanted: 36 days

The median time between the date of first and the last PCR-based detection of SARS-CoV-2 infection was 40.5 days (1.Q.–3.Q.: 26.5–66), the time to the first negative sample was 59 days (1.Q.–3.Q.: 42–164.5). The time to the last positive test was shorter in the organ-transplanted subgroup compared to patients with B cell depletion (median 36 vs. 41 days, respectively) but varied greatly between patients in general (see Fig. 1).

**Fig. 2:**
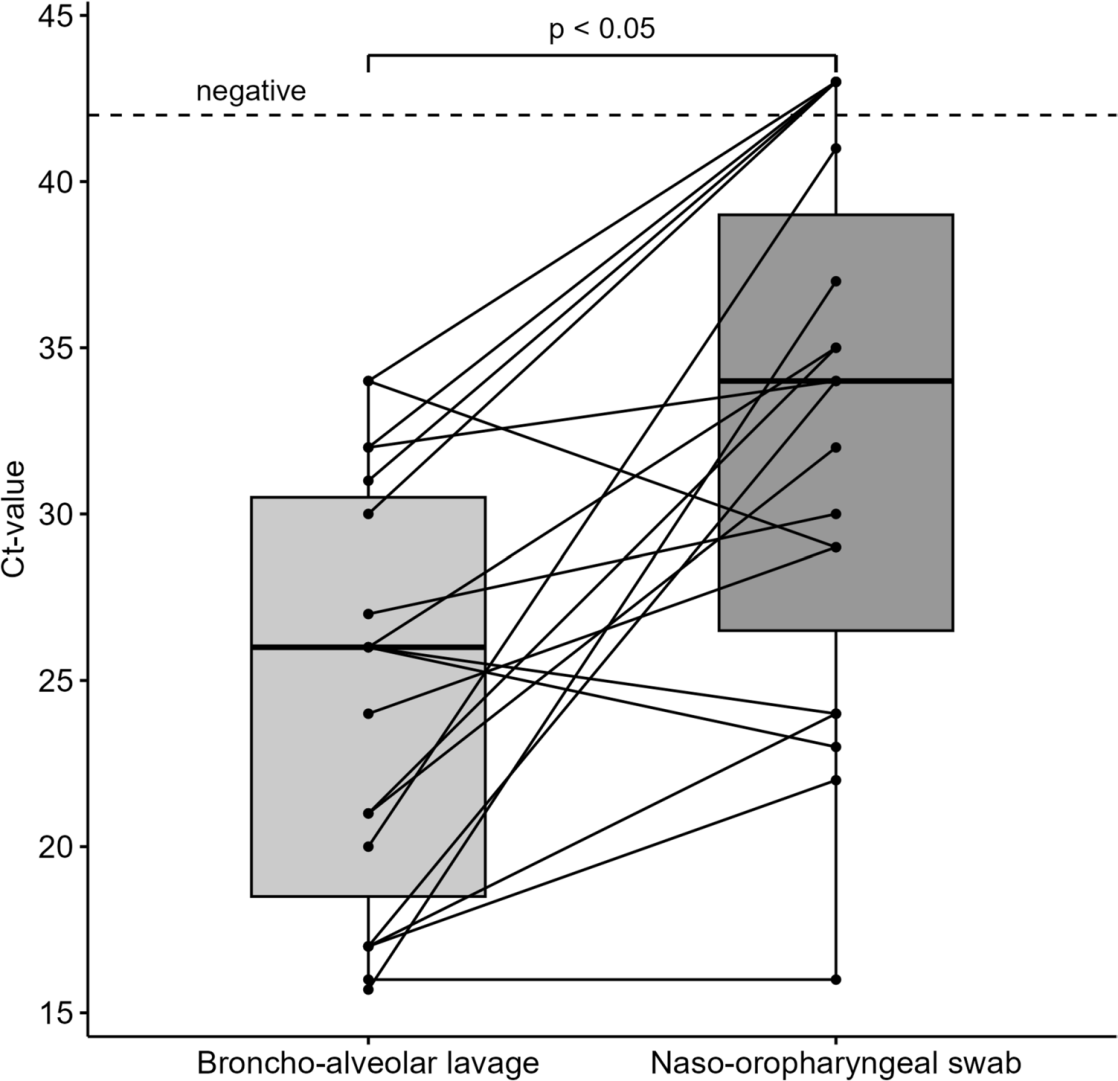
Differences in viral load detection according to sample type by patient. Differences in detected viral load according to sampling location in patients with a positive broncho-alveolar lavage for SARS-CoV-2. Naso-oropharyngeal swabs were paired according to sampling date ± 2 days before and after the date of broncho-alveolar lavage. Significance test was performed using paired Wilcoxon test with exact distribution. The dashed line indicates the limit of detection. Abbreviation: *Ct* Cycle of threshold

In patients with samples taken from the upper and the lower respiratory tracts within a three-day time frame, we found a significant difference of viral load of around eight Ct-value steps between lower respiratory tract samples (median Ct-value 26) and upper respiratory tract samples (median Ct-value 34) (see Fig. 2, *p *= 0.007).

### Radiological findings

**Fig. 3:**
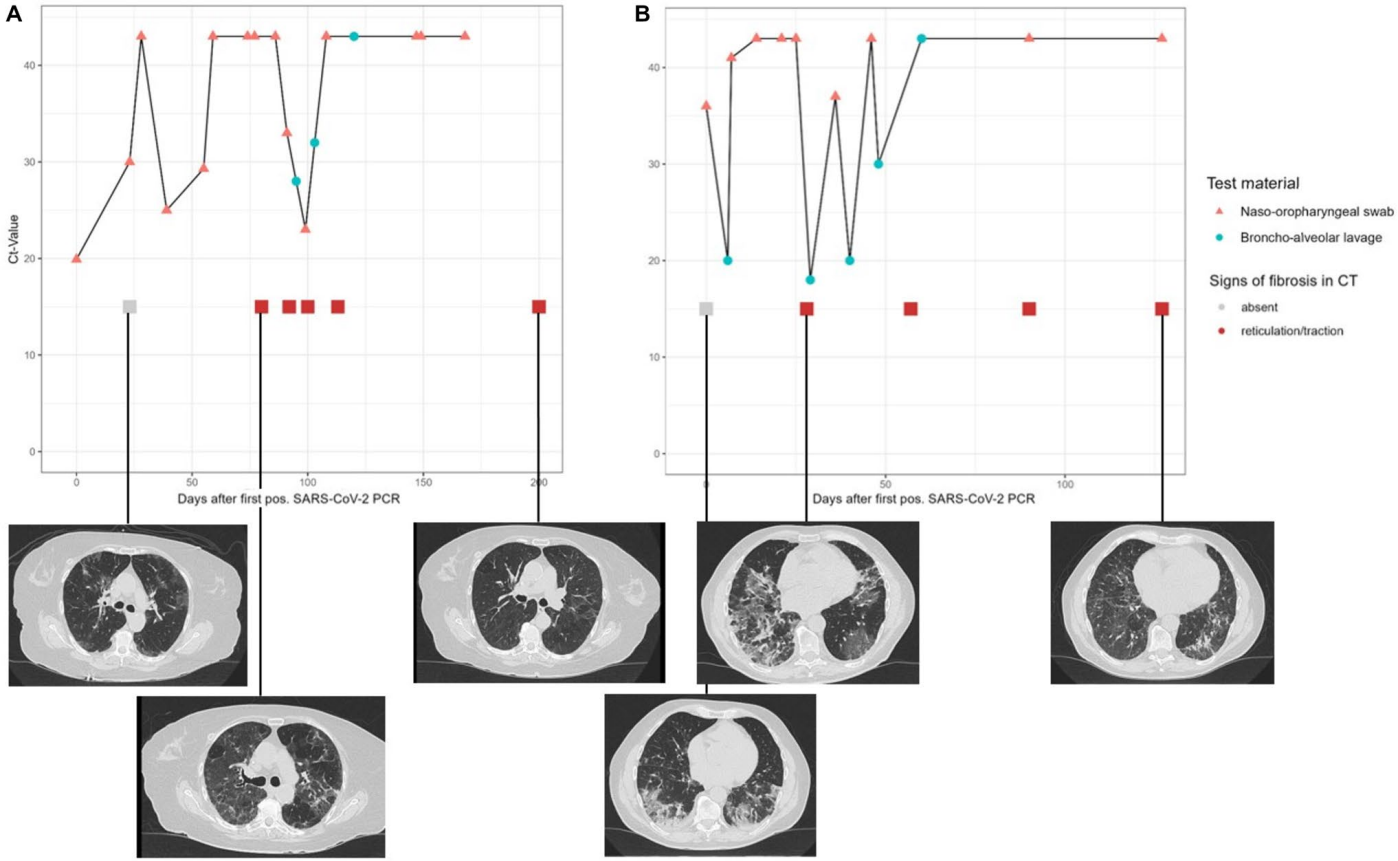
Two exemplary cases of occurrence of radiological changes and the concomitant pattern of SARS-CoV-2 detection during time course. **A** and **B** represent two different exemplary patients with their corresponding thoracic CT scans over time. Notably, patient **A** exhibited considerable regression of fibrotic-like lung changes, whereas patient **B** showed only a limited degree of regression in such change

In 75% of patients (27/36), a CT scan was performed, with the first scan at a median of 14 days (1.Q.–3.Q.: 2–23.5 days) after the first positive test and varying follow-up CTs (75% of CT scans within 114 days after the first positive test). In 7 patients, preexisting pulmonary diseases including pulmonary fibrosis (*n* = 3) and emphysema ( *n* = 3) were prevalent. The findings in the initial CT scan were grouped in 37% (10/27) as RSNA Category 1 and 33% (9/27) as CORADS Category 4/5, indicating a typical pattern of lung involvement by SARS-CoV-2. In the follow-up CT scans, we found newly emerging patterns of fibrotic-like changes in 30% (8/27) of the patients after a median of 50 days (range: 28–193 days) after the first positive test (see Fig. 3 & Supplementary Fig. 2). Patients with new signs of fibrotic-like changes or progression of pre-known lung fibrosis reported initial and recurrent fever, dyspnoea, cough and fatigue in 44% (4/9) cases, whilst only one patient reported no recurrent symptoms (11%, 1/9). Overall, recurrent fever was the most frequent symptom (78%, 7/9), followed by ongoing cough (67%, 6/9) and fatigue (56%, 5/9). The radiological picture during the overall course in patients with evolvement of signs of fibrotic-like changes showed in most cases an organising pneumonia pattern (89%, 8/9) after an initial inflammatory phase. In two out of three patients with pre-existing pulmonary fibrosis, we observed changes that could point towards an acceleration of the preexisting lung fibrosis (see Supplementary Fig. 2). Moreover, we found in two patients the presence of a new SARS-CoV-2-like infiltrate after initial unremarkable radiology (one patient with organ transplantation, one patient with allogeneic stem cell transplantation) or a significant rebound after distinct initial improvement in one patient. New signs of fibrotic-like changes appeared more frequently in the group of patients with B cell deficiency (37%, 7/19), compared to patients with solid organ transplant (12%, 2/17).

### Therapeutic approaches and outcome

The majority of patients (81%, 29/36) received at least one antiviral agent (nirmatrelvir/ritonavir, remdesivir, or molnupiravir), 31% (11/36) received two antiviral agents, either as combination or successively, and 39% (14/36) were treated with a combination of antiviral agent and monoclonal antibody preparations. Sotrovimab was the main monoclonal antibody therapy used (16/36). Antiviral treatment for a five-day course, as suggested in immunocompetent patients, failed to achieve a sustained viral suppression in 76% (16/23) of cases. Repetition of the five-day antiviral course, also with an alternative antiviral substance, was successful in one of five cases (20%). Prolonged antiviral therapy for median 10 days was administered in certain cases (9/36), especially in patients with previous failure of short-course antiviral therapy. In patients treated with a prolonged course of antiviral therapy (> 5 days), a drop in viral load in the collected specimens was detected with a sustained clinical response in 4/9 cases (2/9 clinical treatment success without virological data, 3/9 treatment failure, see Supplementary Fig. 3). Patients with clinical treatment success under prolonged therapy received medication for a longer time (6/9, median = 20.5 days, range: 10–61 days) vs. patients with treatment failure (3/9, median = 10 days, range: 7–10 days). However, in one patient with prolonged antiviral therapy, we recognised recurrent fever or reoccurrence/progress of pulmonary infiltrates even under therapy, with delayed reconvalescence. Time of detectable viral replication after start of antiviral treatment was shorter in patients receiving two antiviral agents (median 15 days) than in patients receiving only one antiviral agent (median 31 days) (see Supplementary Fig. 4).

Overall, all-cause in-hospital mortality rate was 3% (1/36), taken into account that 4 patients had to be excluded because of death before giving consent. Of all 5 intra-hospital deaths, only one was associated with COVID-19 and secondary bacterial superinfection with sepsis and multi-organ failure. Four additional patients died after hospital discharge between onset of infection and May 2023 with all four deaths not being directly related to COVID-19. No additional long-term oxygen therapy had to be initiated. The median time between the onset of symptoms and the last known date alive for all patients was 167.5 days (min = 41 days, 1.Q.–3.Q. = 97.5–313 days).

## Discussion

Main findings of the current study are: first, persistent viral replication over months occurs in immunocompromised patients, also in the Omicron era and especially after B cell depletion. Second, persistent infection may lead to slowly progressing pulmonary changes and should be suspected in immunocompromised patients suffering from recurrent fever, cough, dyspnoea and fatigue. Third, viral replication can be limited to or predominantly occur in the lower respiratory tract and therefore diagnostic specimens only from the upper respiratory may miss it. Fourth, clinical symptoms and radiological changes are at least partially reversible under antiviral therapy with prolonged and combined antiviral treatment being more often associated with sustained clinical improvement than short monotherapy.

Whilst prolonged viral replication of SARS-CoV-2 has already been described for previous variants as well as in case reports for Omicron [2, 22, 23], we would like to highlight the potential of a prolonged COVID-19 course in patients with immunosuppression leading to subacute or chronic complications or sequelae. The relevant number of patients in this monocentric study suggests a higher prevalence and probably underdiagnosed facet of the disease, than case reports in the latest literature may show. In the Omicron era, in which mortality rates are lower than under previous variants, morbidity as leading factor has to be taken more seriously, especially in this vulnerable patient collective. Two previous Italian and Japanese case series investigated the combination of (two) prolonged antiviral agents plus a monoclonal antibody in patients with ongoing viral replication and were able to interrupt SARS-CoV-2 replication effectively, even if long-term follow-up data on these patients are still missing [24, 25]. We need further studies to derive the narrowest and still effective therapy regimen.

Unlike previous variants, the Omicron variant of SARS-CoV-2 predominantly replicates in the upper airways [26, 27]. Different from this, we found a shift to the lower respiratory tract in some patients, with loss of detectable viral RNA in the upper respiratory tract. Previous studies have highlighted the possibility of intra-host evolution with emergence of variants especially in immunocompromised hosts that might be adapted to antiviral treatment [28–30]. Whether a change in tropism of the virus in our cases was the reason for the observed shift in the replication site is not yet clear and has to be further investigated. Also the emergence of antiviral resistance mechanisms should be taken into account [31].

In immunocompetent hosts, pulmonary inflammation is induced in the first two to three weeks of SARS-CoV-2 infection which eventually leads to scarring and chronic changes thereafter [32]. In contrast, in immunocompromised hosts, our data suggest subacute progressive pulmonary changes by prolonged or ongoing SARS-CoV-2 replication. The mechanism behind the higher frequency of fibrotic-like lung changes in patients with B cell depletion compared to patients with organ transplantation is not yet clear. The more intense and T cell involving immunosuppression in patients with organ transplantation may suppress inflammatory processes, which can lead to evolvement of fibrotic changes. Anti-fibrotic effects have for instance been discussed for mycophenolate mofetil [33]. This would suggest that at least a relevant part of the observed lung changes is linked to immunological effects, not viral replication itself, as seen for COVID-19 in immunocompetent patients [34, 35]. Another explanation might be that patients with B cell-depleting therapies have underlying diseases that predispose to interstitial lung diseases (e.g. ANCA-associated vasculitis, scleroderma) or have therapeutic regimens with drugs/radiation therapy that have pulmonary toxicities rendering the lung more vulnerable to virus-induced changes. Further radiological characterisation is necessary to review this, especially in the light of changes by the emergence of the Omicron variant.

### Strengths

Our study has several strengths. First, we present a relevant number of immunocompromised patients with ongoing viral replication. A significant proportion of the patients in our cohort had follow-up CT scans of the lung making an assessment of the time course of the occurrence and recurrence of lung changes feasible. In addition, many follow-up SARS-CoV-2 PCR test results were available, especially paired samples between lower and upper respiratory tract, enabling a more detailed analysis of the special risk group of immunocompromised patients.

Furthermore, for many patients, long-term follow-up data were available with a median time of 167 days. This is especially important as we observed relapses of SARS-CoV-2 replication, which could be missed if the follow-up time is too short.

### Limitations

Limitations of our study (apart from those inherent to the retrospective observational design) include the absence of a control group. Therefore, the estimation of the relative risk of ongoing replication or the development of chronic lung conditions is not assessable. The same holds true for the impact of antiviral therapy on the replication pattern and the course of fibrotic lung changes. A selection bias probably results from the exclusion of patients that died before giving consent and the recruitment via our ID consultation service. Deaths of all patients who died after giving consent were considered not COVID-19-related. Whether the previous ongoing replication of SARS-CoV-2 played a role in a reduction of health in general and thereby leading to acceleration of comorbid conditions is difficult to extrapolate.

Second, we tried to describe the time course of radiological changes in our patients based on the repeated CT scans. Still, the exact time point of the occurrence of changes is hard to identify because of a varying frequency of radiological imaging.

Third, we only included patients with severe immunosuppression like solid organ transplant recipients or B cell-depleted patients which make up the majority of patients with ongoing SARS-CoV-2 replication in our tertiary care setting. Patients with other types or levels of immunosuppression could also be affected by prolonged SARS-CoV-2-replication.

Fourth, the reporting of symptoms is highly subjective (besides fever). The investigated population at the same time was highly comorbid with multiple other possible causes of fever, cough or fatigue making the identification of COVID-19-related symptoms challenging. Nevertheless, to reduce over-reporting of unrelated symptoms, a thorough individual case review was performed and symptoms were only considered SARS-CoV-2-related, if no other plausible condition was identified.

### Implications

Our results have implications on daily practice. In patients with substantial immunosuppression (especially B cell depletion) reporting prolonged symptoms like fever, cough or dyspnoea, persistent SARS-CoV-2 infection should be considered.

To exclude a suspected prolonged replication, lower respiratory tract samples are essential, as SARS-CoV-2 replication may be low or absent in upper airways. A high delta of viral load between the lower and the upper respiratory tract could help us to consolidate the diagnosis of ongoing replication of SARS-CoV-2 in the lower respiratory tract.

Continuous detection of a high viral load (Ct-value < 25) in respiratory material especially from the lower respiratory tract should be taken seriously. Further radiological (re-)imaging should be initiated to detect or exclude chronic lung changes, even with an initial unremarkable lung imaging, as these pulmonary changes may develop over time.

As many patients especially after B cell depletion fail to mount an effective immunity against SARS-CoV-2, antiviral therapy is a major part of most therapeutic approaches towards persistent SARS-CoV-2 infection. Therefore, it should be considered at any point in time of infection. Our data suggest that treatment is most effective in combination and over a prolonged period of time. As rebound of infection is possible after discontinuing antiviral treatment, follow-up testing is essential. The effect of antiviral treatment on chronic pulmonary changes is not yet conclusive. However, we identified some cases with a pronounced regression of pulmonary changes in CT scans after initiation of antiviral therapy, which was accompanied by clinical improvement.

## Conclusion

With the rise of the Omicron variant, we see a shift in the clinical course of infection in immunocompromised patients from fulminant towards subacute prolonged infection. Recurrent fever, cough, dyspnoea and fatigue may be unspecific indicators of ongoing SARS-CoV-2 replication. The diagnostic work-up should include pulmonary imaging and virological samples from the lower respiratory tract as persistent replication predominantly may occur in this compartment and may lead to progressive fibrotic changes of the lung. Most patients under substantial immunosuppression fail to mount a prompt and robust immunity against SARS-CoV-2. Therefore, antiviral treatment is key to most therapeutic approaches. Our data may point towards prolonged treatment duration and a possible benefit of a combination of antiviral agents, which may result in sustained termination of SARS-CoV-2 replication, clinical improvement as well as regression of inflammatory and fibrotic pulmonary changes.

### Supplementary Information

Below is the link to the electronic supplementary material.

Supplementary file1 (DOCX 987 KB)

## Data Availability

Anonymized data are available upon request to the corresponding author.
