# Supplementary material for: Clinical phenotype and outcome of persistent SARS-CoV-2 replication in immunocompromised hosts: a retrospective observational study in the Omicron era
Source: Infection. 2023 Dec 14;52(3):923–33. doi: 10.1007/s15010-023-02138-0 (PMC11142974; doi:10.1007/s15010-023-02138-0)
Supplement: Supplementary file 1 — Supplementary file1 (DOCX 987 KB) [file 15010_2023_2138_MOESM1_ESM.docx]

**Supplementary**

Supplementary Figure 1: STROBE flow chart of patient inclusion


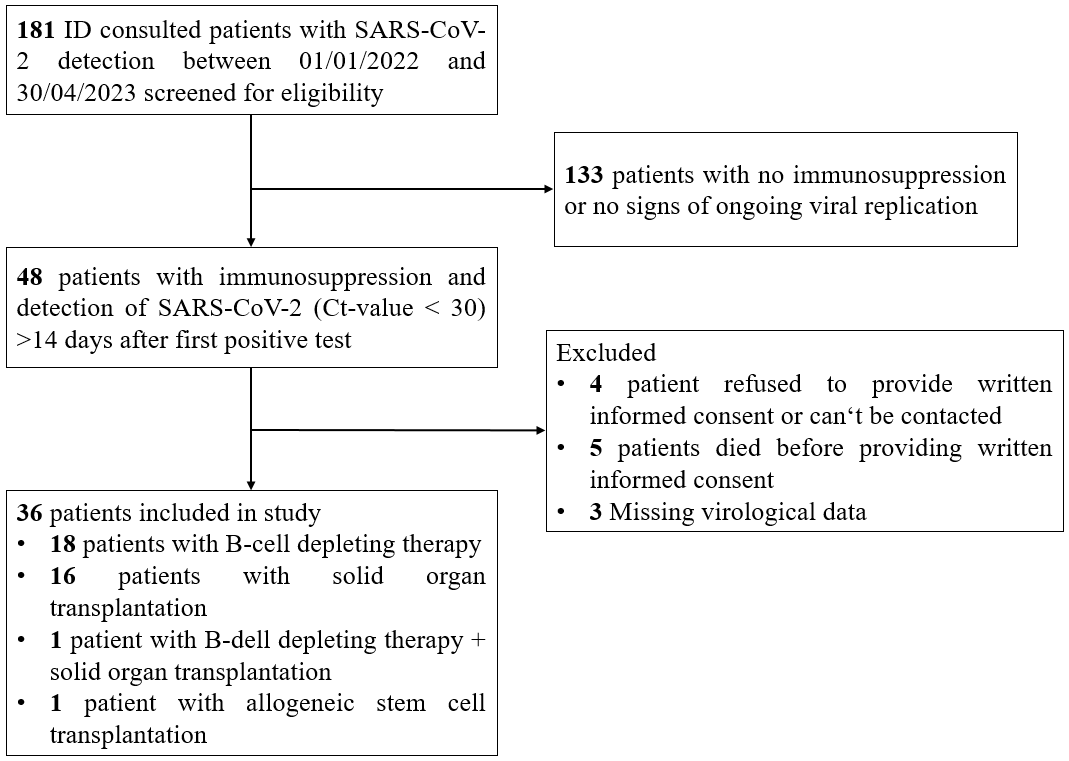


Supplementary Figure 2: Occurrence of radiological changes over time of SARS-CoV-2 infection in patients with repeated CT scans (n = 20)


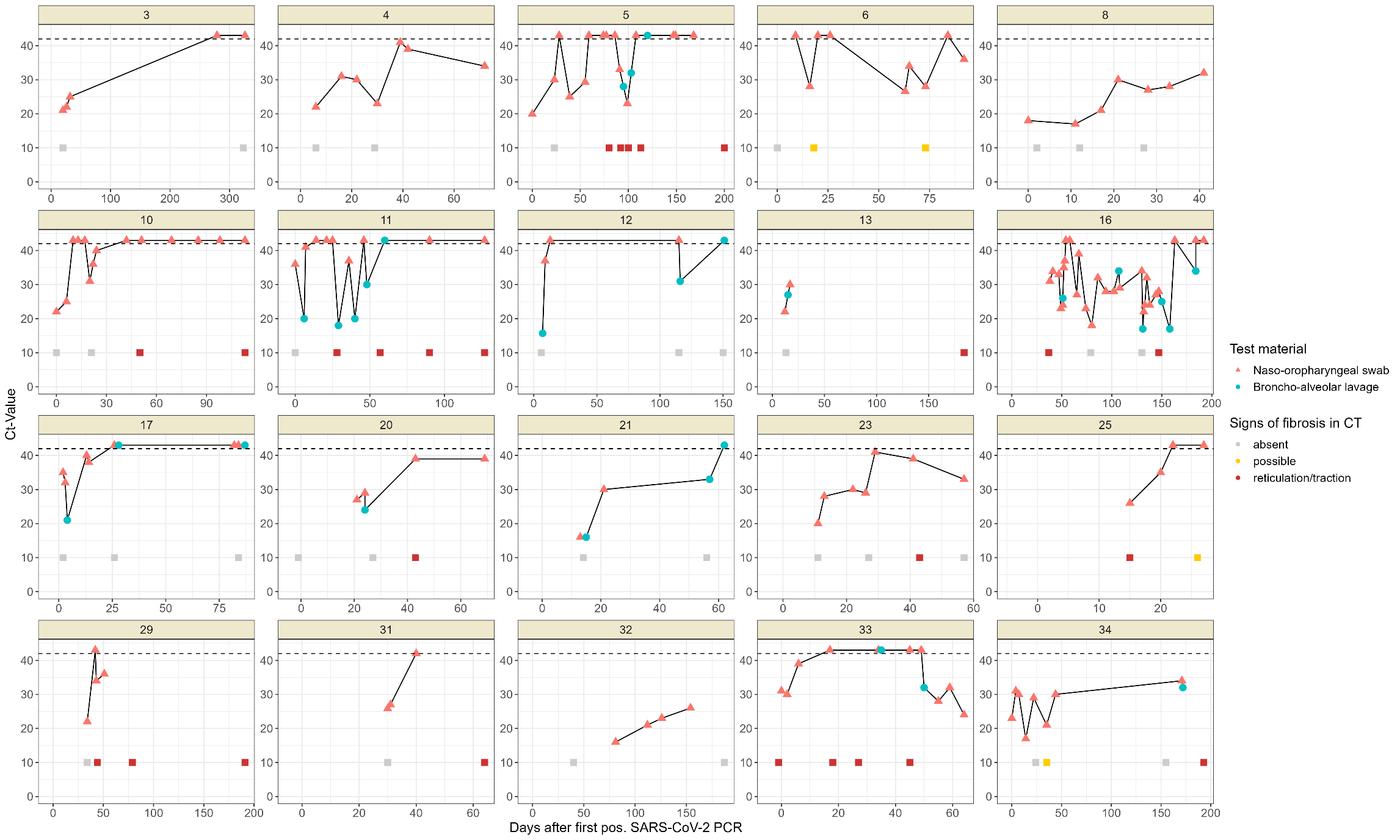


Dashed line represents the cut-off for a negative swab result (Ct-value > 41). At y = 10, results of CT scans at this time point coloured according to findings of signs of fibrosis were shown.

Supplementary Figure 3: Patient-wise (n = 36) overview of administered antiviral therapy and the effect on detected SARS-CoV-2 viral load in respiratory specimen


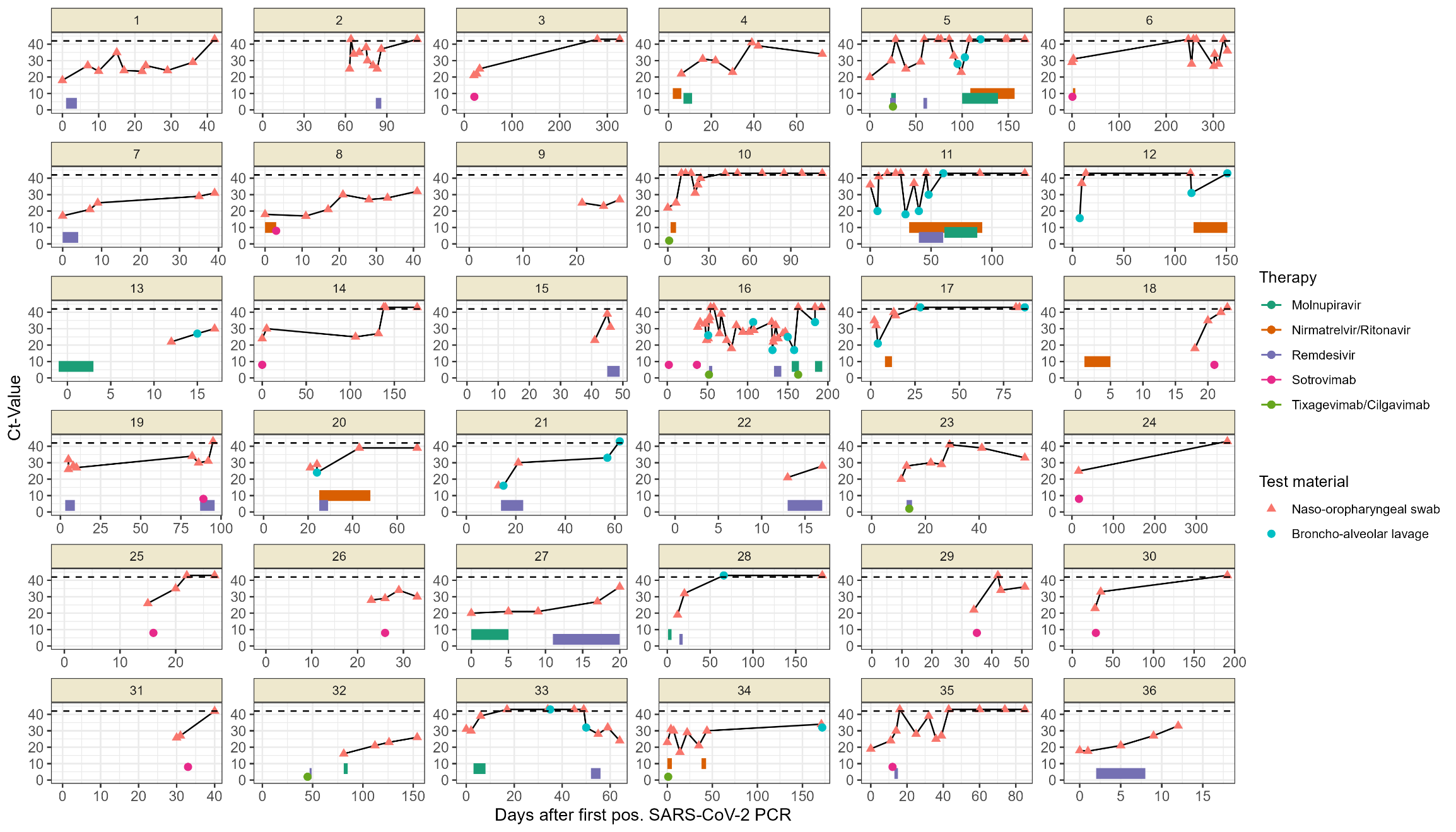


The dashed line represents the Cut-off for a negative swab result (Ct-value > 41).

Supplementary Figure 4: Time of SARS-CoV-2 RNA detection/positivity in respiratory specimen after stop of antiviral therapy with one or more antiviral agents

Supplementary Figure 5: Anti-S1-antibody levels of immunocompromised patients prior to infection

Patients which received Tixagevimab/Cilgavimab as prophylaxis were excluded.

Supplementary Table 1 Distribution of radiological changes in the initial and the first follow-up CT scan

| **Predominant radiological pattern** | **Initial CT scan (n = 27)** | **First follow-up CT scan (n = 20)** |
| --- | --- | --- |
| No pattern | 4 (15%) | 3 (15%) |
| Ground-glass opacities | 13 (48%) | 6 (30%) |
| Consolidation | 5 (19%) | 5 (25%) |
| Mixed ground-glass opacities/consolidation | 5 (19%) | 5 (25%) |
| Fibrotic reticulation | 0 (0%) | 1 (5%) |
